# Gut dysbiosis induced by sunflower oil-based sucrose-free high-fat diet leads to steatohepatitis

**DOI:** 10.3389/fnut.2026.1831803

**Published:** 2026-09-11

**Authors:** Fatemah Bahman, Md. Zubbair Malik, Shihab Kochumon, Rasheeba Nazim, Ashraf Al Madhoun, Sardar Sindhu, Jaakko Tuomilehto, Fahd Al-Mulla, Rasheed Ahmad

**Affiliations:** 1 Department of Immunology & Microbiology, Dasman Diabetes Institute, Dasman, Kuwait; 2 Translational Research, Dasman Diabetes Institute, Dasman, Kuwait; 3 Department of Public Health, University of Helsinki, Helsinki, Finland; 4 Department of Public Health and Welfare, Finnish Institute for Health and Welfare, Helsinki, Finland

**Keywords:** fish oil, gut dysbiosis, hepatic inflammation & steatosis, high fat diet, sunflower oil

## Abstract

High-fat diets (HFDs) are known to disrupt gut microbiota, contributing to obesity, inflammation, and metabolic disorders. Although sucrose is a known driver of gut dysbiosis, the microbiome alterations caused by sucrose-free fish oil and sunflower oil-based HFDs remain unclear. To investigate how sucrose-free sunflower oil-based (S-HFD) and fish oil-based (F-HFD) high-fat diets influence gut microbiota composition, metabolic health, and liver inflammation in mice. C57BL/6 mice were fed either S-HFD or F-HFD for 24 weeks. Body weight, insulin sensitivity, liver inflammation, and gene expression were assessed. Gut microbiota composition was analyzed using 16S rRNA sequencing, followed by diversity analysis and taxonomic profiling with Microbiome Analyst and MIAOME tools. Despite similar body weights between groups, the gut microbiota composition differed significantly. The S-HFD group showed a higher abundance of Firmicutes (40%) compared to the F-HFD group (3%), while Verrucomicrobia were dominant in F-HFD (26%) and nearly absent in S-HFD. Taxa such as RF39, Christensenellaceae, Mogibacteriaceae, and Yaniella were enriched in S-HFD mice and associated with metabolic and immune dysregulation. S-HFD mice also had elevated fasting glucose, increased hepatic monocyte/macrophage (F4/80+) infiltration, macrovesicular steatosis, lobular inflammation, and upregulation of genes related to fatty acid oxidation (Cpt1a), monocyte chemotaxis (Ccl2), lipogenesis (Scd1, Fasn, Acaca), and glycolysis (Pklr). Conversely, F-HFD mice showed increased expression of the insulin-sensitive gene FATP1. Sucrose-free sunflower oil- and fish oil-based high-fat diets induce distinct gut microbiota changes and metabolic responses. S-HFD is associated with gut dysbiosis and steatohepatitis-like features, highlighting the importance of fat sources in shaping microbiome-host interactions in metabolic disease.

## Graphical abstract

**Figure:**
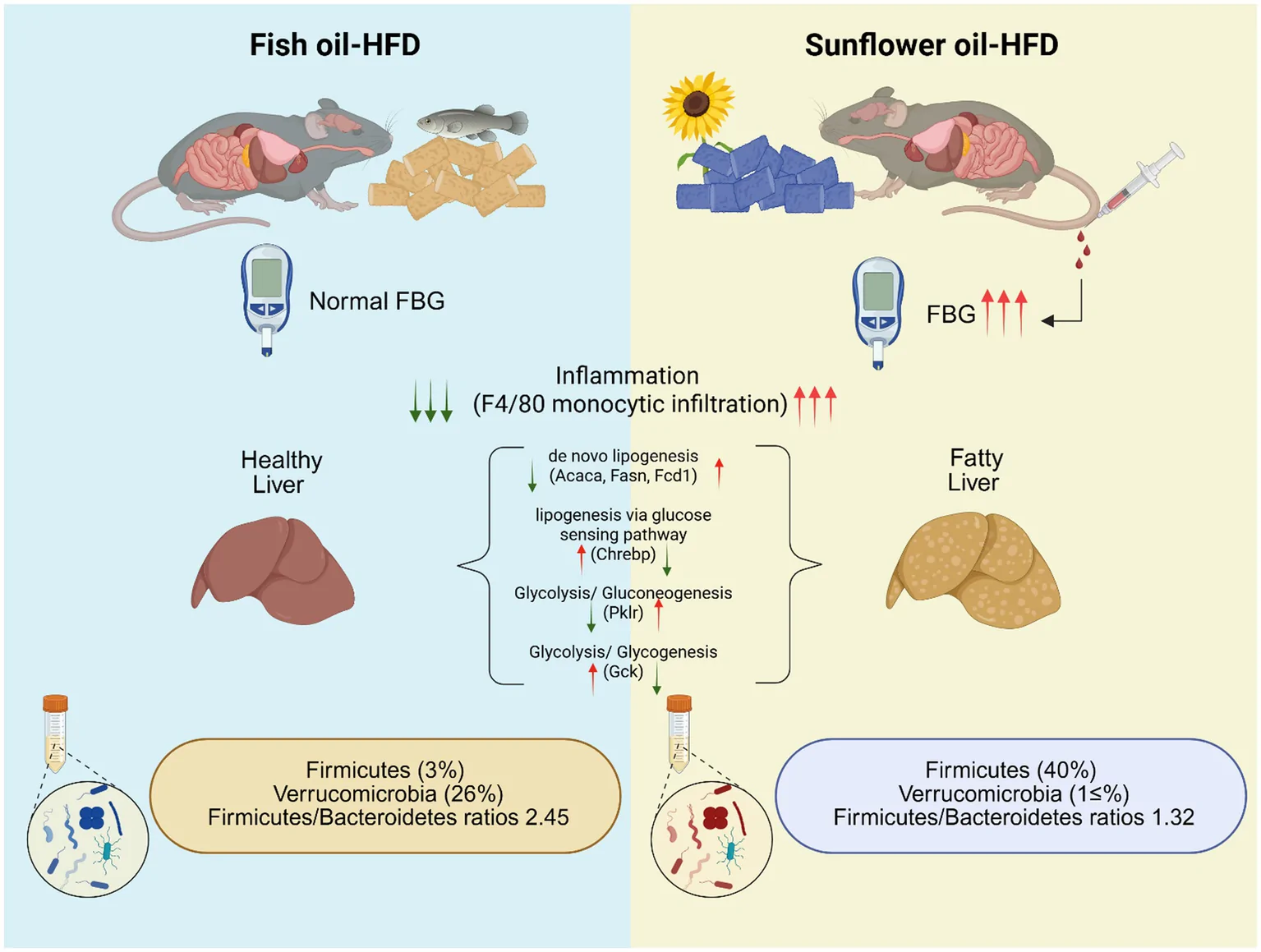

## 1 Introduction

Obesity is characterized by an excessive or abnormal accumulation of body fat that can impair overall health. In recent decades, it has become a global public health concern, with its prevalence rising steadily (1, 2). Obesity is a well-established risk factor for numerous chronic and life-threatening conditions, including diabetes (3, 4), cancers (5, 6), cardiovascular disease (7, 8), polycystic ovary syndrome (9) and hypertension (10–13).

Energy-dense diets rich in saturated fats and glucose play a key role in the development of obesity and insulin resistance. Commonly consumed vegetable oils vary by region, with olive oil being prevalent in the Mediterranean, palm and coconut oils in Asia, soybean oil in America, and sunflower oil primarily consumed in Eastern Europe and North Africa (14, 15). Nonetheless, the source and quality of dietary fat play a crucial role in preventing metabolic disorders. The primary distinction between the different dietary oils is in their fatty acid composition, which is saturated fatty acids (SFAs) in coconut oil, polyunsaturated fatty acids (PUFAs) in sunflower oil and monounsaturated fatty acids (MUFAs) in olive oil (16, 17). The overall composition of dietary fatty acids can significantly influence lipid metabolism and inflammation (18–22). Sunflower oil is composed of approximately 15% saturated fatty acids and 85% unsaturated fatty acids, with oleic acid ranging from 14 to 43% and linoleic acid comprising 44–75% (23). Although sunflower oil improved the plasma lipid profile, particularly by reducing circulating triacylglycerol levels, it did not prevent or attenuate high-fat diet-induced insulin resistance and inflammation (24). On the other hand, fish oil consists cholesterol and omega-3 long-chain polyunsaturated fatty acids (PUFAs), including such as docosahexaenoic acid (DHA) and eicosapentaenoic acid (EPA), which are recognized for their anti-inflammatory and anti-cancer properties and cardioprotective (25, 26). Different types of diet play a crucial role in influencing the function and composition of the gut microbiome. Dietary fats influence gut microbial activity, thereby affecting the body’s ability to metabolize various nutrients (27–29). A high-fat diet and saturated fats influence gut microbiota diversity (30). These studies led to the recommendation that a high-fat diet and consumption of saturated fats should be avoided, and a high intake of monounsaturated (MUFA) and polyunsaturated (PUFA) omega-3 encouraged, to positively influence gut microbiota and limit inflammation (31–34). Several dietary and environmental factors can contribute to promoting the proliferation, gut dysbiosis of pathogenic bacteria. Various dietary and environmental factors can lead to the growth of harmful bacteria and cause gut dysbiosis. These harmful bacteria can then cause chronic inflammation and lead to the development and progression of various inflammatory and metabolic disorders, including metabolic dysfunction associated steatohepatitis (MASH) (35, 36), metabolic-associated steatotic liver disease (MASLD) (37), inflammatory bowel diseases (IBD) (38), obesity (39, 40) and type 2 diabetes (41, 42).

High-fat diets (HFDs) commonly promote weight gain, hepatic steatosis, and increased hepatic inflammatory cytokine expression, effects that are often attributed to their combined high fat and sucrose content (89). However, accumulating evidence indicates that dietary fat sources differ markedly in their ability to shape obesity-related outcomes and gut microbiome composition (90–93). These differential effects may be partly explained by variations in saturated fatty acid (SFA) and unsaturated fatty acid (UFA) composition (94). Although fish oil and sunflower oil are often considered healthier dietary fat sources, they exert distinct effects on the gut microbiome. Therefore, to evaluate the effects of dietary fat quality independent of sucrose, we used sucrose-free high-fat diet models enriched with sunflower oil or fish oil. These models allowed us to examine how specific dietary fat sources influence gut microbiome remodeling and how these changes relate to hepatic steatosis, inflammation, fibrosis, systemic glucose tolerance, and insulin resistance. Our previous study revealed that a high-fat diet enriched with soybean oil negatively impacts gut health and induces dysbiosis, whereas a F-HFD did not cause gut microbiota imbalances in mice. Furthermore, a strong positive correlation was observed between gut microbial dysbiosis and inflammatory pathways, as mice fed a soybean oil-based high-fat diet exhibited lobular inflammation, fibrosis, and macrophage accumulation in the liver, whereas those on a fish oil-based high-fat diet did not develop such features (20).

Additionally, we also reported that mice fed a Cocoa-HFD exhibited elevated gene expression of inflammatory markers such as Interleukin (IL-12), C-C motif chemokine ligand 2 (Ccl2), and Tumor necrosis factor alpha (TNF-α, along with a greater risk for liver fibrosis when compared to those on Fish-HFD (21). The gut microbiota is now seen as an important focus for diet and treatment plans to help prevent and manage inflammation and metabolic disorders.

Incorporating fish oil into a low-fat diet significantly altered the host intestinal microbiota, promoting an increase in Bifidobacterium species from the phylum Actinobacteria (43). Fish diet was also shown to positively shape the host microbial ecosystem (25, 44, 45). Although these diets may have different nutritional effects, no studies have compared the impact of a sucrose-free S-HFD and a sucrose-free F-HFD on gut dysbiosis and its link to liver steatosis. In this study, two different dietary fats, namely sunflower oil and fish oil, were used to determine the effects of these dietary fats on the dysbiosis of the gut microbiota in mice fed for a period of 24 weeks. We also examined peripheral insulin sensitivity, glucose tolerance, low-grade inflammation and hepatic steatosis.

## 2 Materials and methods

### 2.1 Animals

All animal work followed the National Institutes of Health guidelines for caring for lab animals and was approved by the ethics committee (Approval No. RA AM 2016–007). C57BL/6 mice from Jackson Laboratory were kept and bred at the Animal Core Facility of Dasman Diabetes Institute. The animal was fed a standard chow diet with free access to food and water and is kept under a 12-h cycle of light and dark. Experimental procedures were conducted on male mice aged 8 to 10 weeks. Animals were randomly assigned into two groups, each consisting of 5–6 mice. Two sucrose-free high-fat diets, each providing 45% of total energy from fat, were used: a sunflower oil-based diet (D18060405) and a fish oil-based diet (D18060407), both from Research Diets Inc. Body weight and food consumption were monitored weekly throughout the study. On weeks 21 and 22, oral glucose tolerance tests (OGTT) and insulin tolerance tests (ITT) were performed, respectively. Following 24 weeks of dietary intervention, mice were sacrificed, and various organs and tissues were collected. Samples were immediately snap-frozen in liquid nitrogen and stored at −80 °C for further analysis. Blood samples were collected for plasma isolation, which was also stored at −80 °C. For histological examination, liver tissues were fixed and embedded in paraffin. We collected and preserved fecal samples from each mouse at −80 °C until microbiome analysis is performed.

### 2.2 Insulin tolerance test and oral glucose tolerance test

The ITT was performed at the beginning of the 22 weeks of feeding with F-HFD or S-HFD, with mice fasted for 4 h prior to the procedure. Insulin (0.75 U/kg body weight) was administered intraperitoneally, and blood glucose levels were measured at 0, 15, 30, 45, 60, and 90 min (46). The OGTT was conducted in mice at at the beginning of the 21 weeks of feeding, following a 12-h fast. Each mouse was administered 1 g/kg body weight of glucose orally, and blood glucose levels were measured at multiple time points: 0, 15, 30, 45, 60, 90, 120, 150, and 180 min (46).

### 2.3 Plasma measurements

Plasma concentrations of key metabolic hormones such as insulin, PYY, C-peptide, amylin, glucagon, leptin, and pancreatic polypeptide (PP) were quantified using a 15-plex multiplex assay (Cat. # MMHE-44 K, MILLIPLEX Mouse Metabolic Hormone Expanded Panel, Millipore, Burlington, MA, USA) following the manufacturer’s instructions.

### 2.4 Histopathological examination

F4/80 immunohistochemistry (IHC) staining was performed on 4 μm-thick paraffin-embedded liver sections. Tissues were first deparaffinized with xylene and rehydrated through graded ethanol (100, 95, 75%) into distilled water. Antigen retrieval was done by heating slides in a citrate buffer (pH 6.0, Dako) using a pressure cooker for 8 min, then cooling for 15 min at 25 °C. After rinsing with PBS, the tissue sections were treated with 3% hydrogen peroxide for 30 min to block endogenous peroxidase activity. To minimize non-specific binding, the slides were incubated with 5% non-fat milk for 1 h, followed by a second blocking step using 1% bovine serum albumin (BSA) for an additional hour. The sections were then incubated overnight at 25 °C with a rabbit polyclonal anti-F4/80 antibody (1:100, Abcam ab100790). After washing with PBS containing Tween-20, the slides were incubated for 1 h with an HRP-conjugated goat anti-rabbit secondary antibody from the EnVision Kit (Dako). The signal was developed with DAB substrate, producing a brown color. Slides were rinsed, counterstained with Harris hematoxylin, then dehydrated with increasing ethanol concentrations (75, 95, 100%), cleared in xylene, and mounted using DPX medium. Images were captured at 20 × magnification using a PanoramicScan II slide scanner (3DHISTECH). Ten different areas per tissue section were selected for analysis. Stained regions were annotated in Aperio ImageScope software, and quantification was performed using its Positive Pixel Count algorithm (version 9). The number of positive pixels was normalized to the total area to account for size differences. Color thresholds were kept consistent across all slides, and final color overlays were visually checked for accuracy.

Additionally, liver and adipose tissues were stained with hematoxylin and eosin (H&E) to assess general histology, and with Oil Red O to examine fat accumulation, using standard lab protocols (47, 48).

### 2.5 Gene expression analysis by qRT-PCR

Complementary DNA (cDNA) was synthesized from 1 μg of total RNA using the High-Capacity cDNA Reverse Transcription Kit (Thermo Scientific, Cat. #4368814), following the manufacturer’s instructions. Quantitative real-time PCR was carried out using the QuantStudio™ 5 Real-Time PCR System with gene-specific TaqMan probes and TaqMan master mix (Thermo Scientific, Cat. #4369016). All reactions were run in triplicate under standard thermal cycling conditions. Gene expression levels were normalized to Gapdh using the $2^{-\Delta \Delta \mathrm{ct}}$ method. Details of all TaqMan primer/probe sets are listed in Supplementary Table S1.

### 2.6 Microbiome sequencing

Genomic DNA was extracted from mouse fecal samples using the QIAamp DNA Fast Stool Mini Kit (Qiagen, Germany), following the manufacturer’s instructions. DNA concentration was measured with a Qubit fluorometer (Thermo Fisher Scientific, USA). Bacterial 16S rRNA genes (V3–V4 regions) were amplified from microbial DNA (5 ng/μL) using specific primers with adapter overhangs (Supplementary Table S2). PCR amplification was done using the KAPA HiFi HotStart ReadyMix PCR Kit (Roche Diagnostics). PCR products were checked with a Bioanalyzer (Agilent Technologies) using a High Sensitivity DNA chip, cleaned using AMPure XP beads, and indexed with the Nextera XT Index Kit (Illumina Inc.). The libraries were then purified, normalized, combined (up to 24 samples), and sequenced using paired end reads on the Illumina MiSeq platform.

### 2.7 16S rRNA microbiome analysis and bioinformatics statistics

Sequence data were processed in QIIME 2 (version 2022.8) (49, 50). Primers were removed using q2-cutadapt, and paired-end reads were denoised, merged, and chimera-filtered using DADA2 (51) as implemented in q2-dada2 (denoise-paired), with forward and reverse reads truncated. This procedure resolves amplicon sequence variants (ASVs) at single-nucleotide resolution; no additional OTU clustering was applied. ASVs were taxonomically classified and phylogenetically placed against the Greengenes2 reference (release 2022.10) (52) using (q2-greengenes2 non-v4-16s/a naive Bayes classifier trained on the V3–V4 region). ASVs with fewer than 10 total counts across all samples were removed prior to downstream analysis. Filtered feature tables were imported into Microbiome Analyst (53). Relative abundances were summarized at phylum, class, order, family, genus, and species level; because V3–V4 amplicons afford limited species-level resolution, species-level assignments are reported as tentative. Differential abundance between the S-HFD and F-HFD groups was assessed using the Wilcoxon rank-sum (Mann–Whitney U) test, with *p* values corrected using the Benjamini–Hochberg false discovery rate procedure.

### 2.8 Biodiversity analysis

Intra-sample (alpha) and inter-sample (beta) diversity analyses were conducted to assess microbial diversity (52, 54, 55). Alpha diversity was evaluated using multiple metrics, including Observed species, Chao1, ACE, Shannon index, Simpson index, Fisher’s alpha, and phylogenetic diversity (56), following the methodologies cited in the respective references. These measures provided detailed insights into the richness and evenness of bacterial genera and species within each sample. Beta diversity was assessed using Bray–Curtis dissimilarity and UniFrac distance matrices, both weighted and unweighted, to evaluate differences in microbial composition between samples (52). The distance matrices were shown using two-dimensional Principal Coordinate Analysis (PCoA) plots. To check for significant differences in alpha diversity, we used the Mann–Whitney U and Kruskal–Wallis tests. For beta diversity, we used PERMANOVA to assess group differences.

### 2.9 Identification of biomarker microbiome

LEfSe (Linear Discriminant Analysis Effect Size) was used to find bacterial groups that were significantly more or less abundant between different phenotypic groups. The analysis used a Benjamini–Hochberg FDR correction, with a *p*-value cutoff of 0.05 and an LDA score threshold of 2.0. Bar plots for the LEfSe results were created using the MicrobiomeAnalyst tool (53).

### 2.10 Functional prediction and metabolic pathway analysis of gut microbiota

PICRUSt2 (Phylogenetic Investigation of Communities by Reconstruction of Unobserved States 2) was used to predict the functional capabilities of gut microbiome communities and to assign relevant annotations based on these predictions (57). The predicted functional profiles were further analyzed using the MicrobiomeAnalyst package (53). To explore the metabolic pathways associated with the predicted microbial functions, the MetaCyc database (58) was employed, providing comprehensive information on experimentally validated chemical compounds, reactions, enzymes, and metabolic pathways. Additionally, correlations between the microbial communities and their associated metabolites were assessed using the Microbiome Analyst package.

### 2.11 Prediction of microbiome-epigenome interactions

The human microbiome can affect host gene expression through proteins, components, and metabolites it produces, which can change how genes are regulated and impact overall body function. To predict potential microbiome-epigenome interactions relevant to biomarkers, taxon set enrichment analysis was performed using the MicrobiomeAnalyst platform, along with data from the Microbiome-Host Epigenome (MIAOME) database^1^ (59) and Human Microbe-Disease Association Database (HMDAD)^2^ (60).

## 3 Results

### 3.1 Impact of fish (F-)HFD and sunflower (S-)HFD and diets on gut microbiota diversity and composition

**Figure 1:**
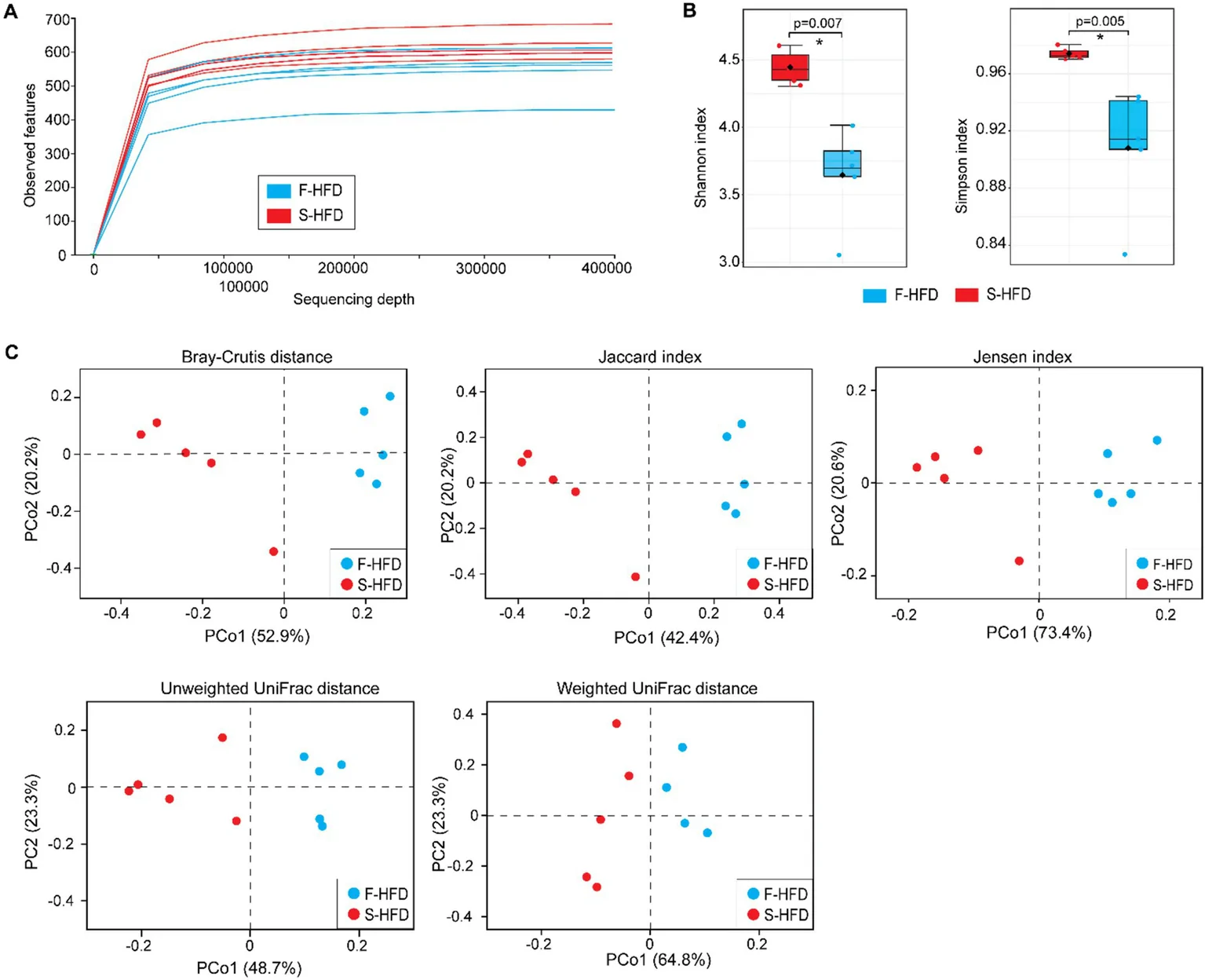
Differential effects of F-HFD and S-HFD diets on gut microbial diversity and composition. **(A)** Rarefaction curves showing the number of observed features (species richness) as a function of sequencing depth in F-HFD (blue) and S-HFD (red) groups. The F-HFD group exhibits microbial richness across all sequencing depths. **(B)** The alpha diversity of gut microbiota in F-HFD and S-HFD fed mice is presented, highlighting variations in microbial diversity and richness between the two diet groups. These differences are depicted using boxplots of Simpson and Shannon indices, which reflect species diversity and evenness within the samples. Mice fed the S-HFD diet exhibited significantly higher diversity compared to those on the F-HFD diet. Statistical significance for these comparisons is indicated by *p* ≤ 0.05. **(C)** Beta-diversity comparisons of the bacterial communities from F-HFD and S-HFD fed mice are shown by PCoA of Bray-Curtis distances, Jensen-Shannon divergence, Jaccard distances, unweighted and weighted UniFrac distances. The percentage of variance accounted for by each principal coordinate is indicated on the respective axis labels.

To examine the effect of F-HFD and S-HFD feeding on gut microbiota composition in mice, gut content samples were analyzed using 16S rRNA V3-V4 gene sequencing with high-throughput Illumina MiSeq technology. This resulted in a total of 5,124,354 reads, with an average of 512,435 reads per sample. Quality filtering procedures removed 20% of reads across all samples, followed by standard quality control assessments to ensure data integrity. Rarefaction curve analysis indicated sufficient sequencing depth to capture the microbial diversity present in the samples (Figure 1A). We examined the differences in microbial richness and community composition between the F-HFD and S-HFD groups in order to evaluate the effects of various high-fat diets on gut microbial diversity. Rarefaction analysis based on observed features demonstrated a consistently higher number of observed taxa in the F-HFD group compared to the S-HFD group across all sequencing depths, indicating greater species richness (Figure 1A). Alpha diversity indices, however, diverged from this pattern, with significantly higher Shannon and Simpson indices in the S-HFD group compared to the F-HFD group (*p* = 0.007 and *p* = 0.005, respectively), a divergence from the richness data that reflects reduced evenness in the F-HFD microbial community (Figure 1B). Beta diversity comparisons further highlighted distinct microbial community structures between the two diet groups. PCoA demonstrated significant clustering by diet using various distance metrics, including Bray–Curtis dissimilarity (ANOSIM, *R*^2^ = 0.47, *p* = 0.015), Jaccard index (ANOSIM, *R*^2^ = 0.76, *p* = 0.02), Jensen-Shannon divergence (ANOSIM, *R*^2^ = 0.45, *p* = 0.012), weighted UniFrac (ANOSIM, *R*^2^ = 0.5, *p* = 0.015), and unweighted UniFrac distances (ANOSIM, *R*^2^ = 0.4, *p* = 0.01) (Figure 1C). These results suggest that S-HFD and F-HFD diets distinctly influence gut microbial diversity and composition in mice.

### 3.2 Altered composition of gut microbiota from mice fed on S-HFD and F-HFD diets

**Figure 2:**
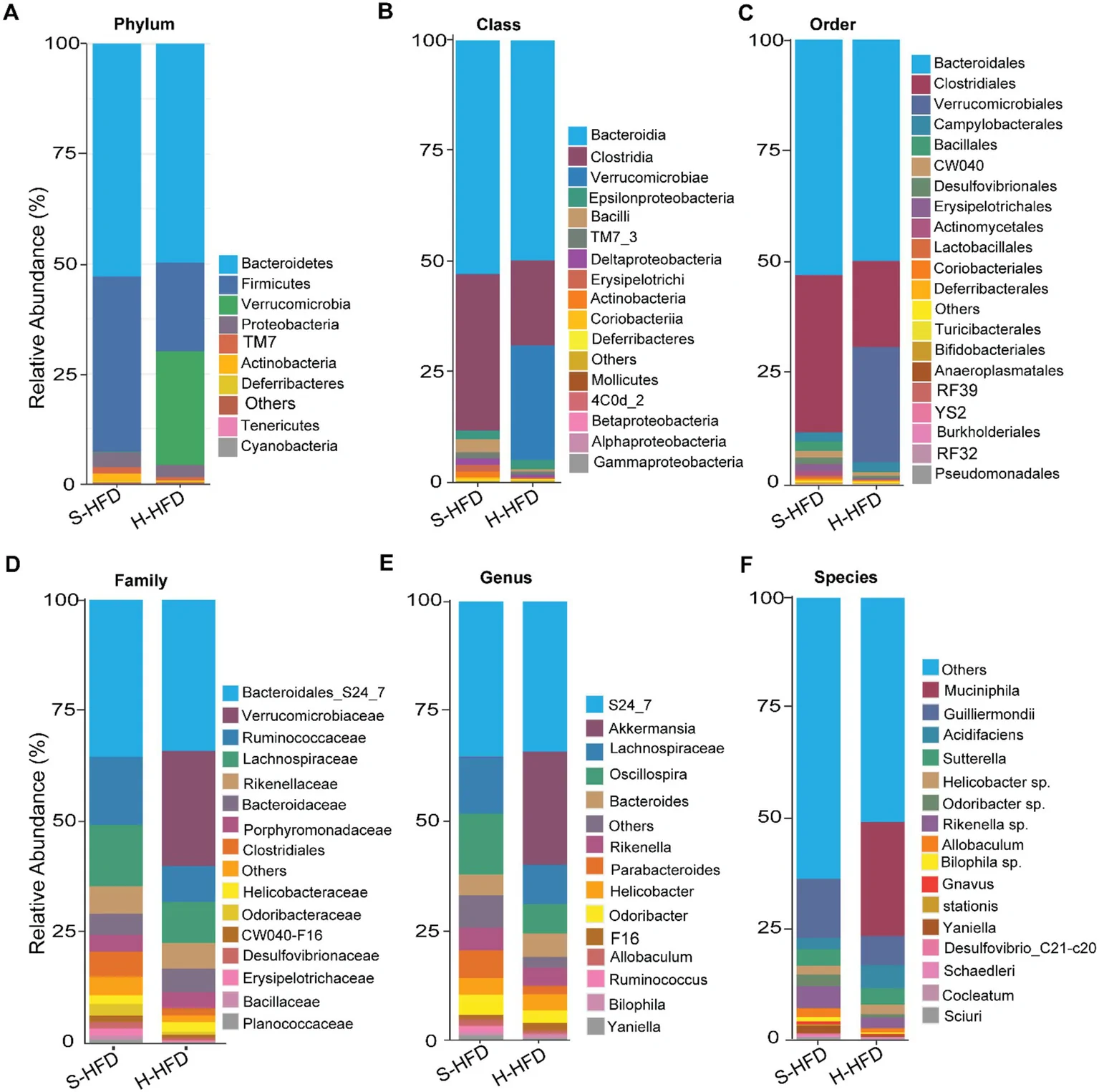
The gut microbiota composition in the F-HFD and S-HFD groups was analyzed at different taxonomic levels. Stacked bar plots display the percentage of bacterial taxa in fecal samples from both groups at the **(A)** phylum, **(B)** class, **(C)** order, **(D)** family, **(E)** genus, and **(F)** species levels. Clear differences in microbial composition were observed between the two diets across all taxonomic levels.

We next examined the differential taxonomic composition of the gut microbiota across various taxonomic ranks. The relative abundance of plots at the phylum, class, order, family, genus, and species levels revealed clear visual distinctions between the S-HFD and F-HFD dietary groups (Figures 2A–F). Furthermore, gut microbiota of mice fed on these diets were mainly composed of the phyla including Firmicutes, Bacteroidetes, Actinobacteria, Verrucomicrobia, Proteobacteria, TM7. As expected, phylum-level composition of gut microbiota was dominated by the taxa including Bacteroidetes (53%), Firmicutes (40%) and Proteobacteria (3%). Interestingly, Firmicutes were detected at higher abundance in mice fed an S-HFD diet (40%) while the abundance of Firmicutes was lower (20%) in mice that were fed on F-HFD. On the contrary, we found that the abundance of Verrucomicrobia was very low (<1%) in mice fed on S-HFD diet while its abundance was high (26%) in mice fed on F-HFD. Also, the Firmicutes/Bacteroidetes (F/B) ratios in S-HFD and F-HFD dietary groups were 1.32 and 2.45, respectively, (Figure 2A). At the class level, Verrucomicrobiae/Clostridia (V/C) ratios regarding the S-HFD and F-HFD groups were 0.02 and 1.33, respectively (Figure 2B). The abundance of 21 dominant OTUs was significantly affected by the S-HFD diet feeding. Most of them belonged to the order *Clostridiales* and their abundance was high in S-HFD fed mice while the order Verrucomicrobiales had very low abundance (<1%) in mice fed on S-HFD, compared with the F-HFD fed group (Figure 2C). Despite the marked inter-individual differences at the family level, gut microbiota abundance was significantly higher for Ruminococcaceae (phylum Firmicutes), Lachnospiraceae (phylum Firmicutes) and Rikenellaceae (phylum Bacteroidetes) in S-HFD fed mice. Among these microbes, Ruminococcaceae/Rikenellaceae (i.e., Firmicutes/Bacteroidetes phyla) ratios for S-HFD and F-HFD dietary groups were 2.46 and 1.39, respectively (Figure 2D). Next, at the genus level, S-HFD group mice had a higher abundance of Oscillospira, Allobaculum, Odoribacter, Parabacteroides and a lower abundance of Akkermansia, Rikenella, and Sutterella as compared to mice of the F-HFD group (Figure 2E). We further analyzed species-level relative abundances and observed that mice in the S-HFD group exhibited higher levels of *Ruminococcus gnavus*, *Staphylococcus sciuri*, *Candida guilliermondii*, and *Clostridium stationis*, along with a notably lower abundance of *Akkermansia muciniphila*, compared to the F-HFD group (Figure 2F).

### 3.3 Differential changes in gut microbiome of mice fed on F-HFD and S-HFD diets

**Figure 3:**
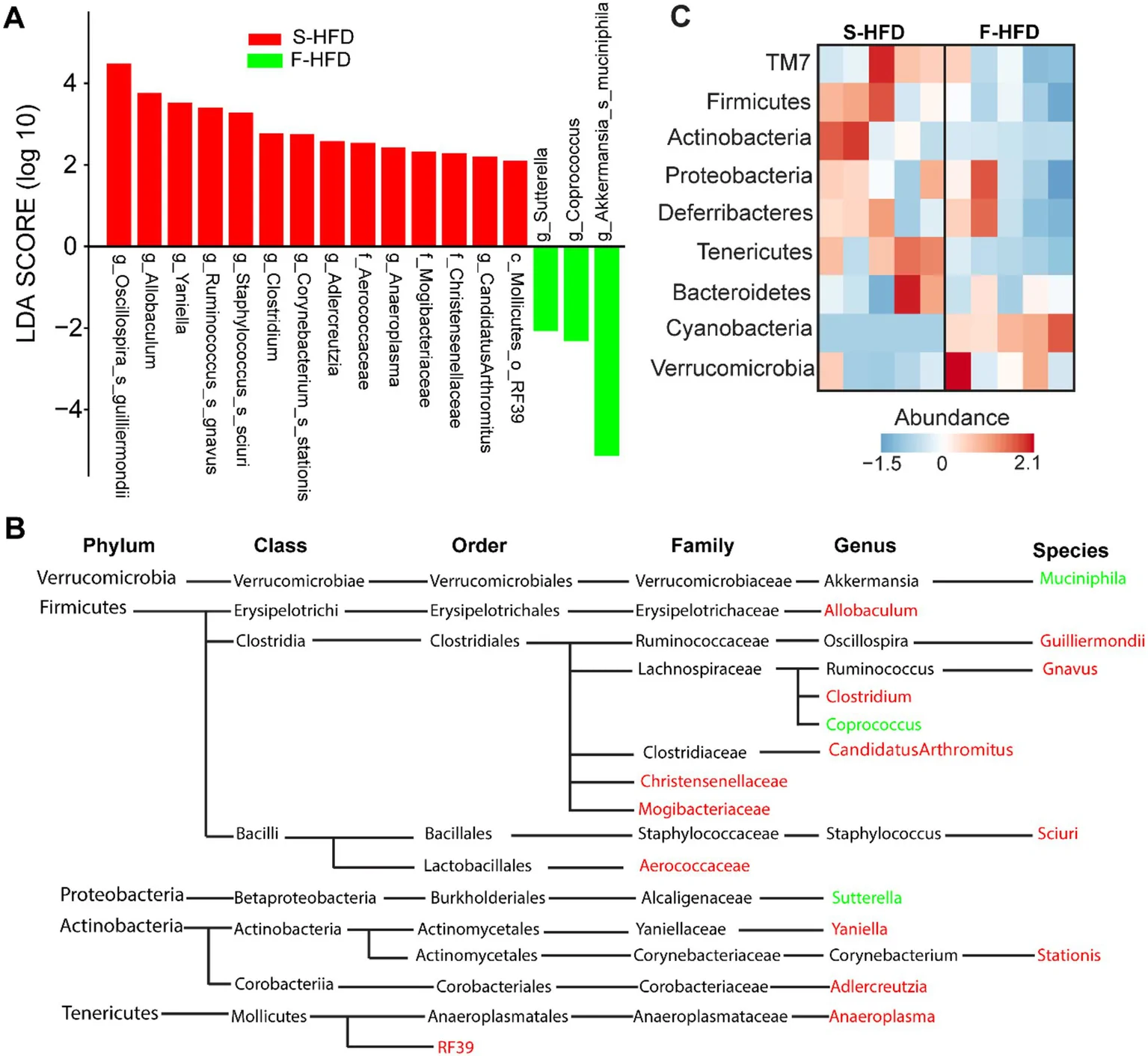
Identification of differentially abundant gut microbiota between S-HFD and F-HFD. **(A)** Histograms of LEfSe score showing higher and lower abundance gut microbiota in S-HFD mice (*n* = 5), compared with F-HFD mice (*n* = 5). The LDA score (log_10_) indicates the effect size of each discriminative feature. Red bars indicate taxa enriched in S-HFD, and green bars indicate taxa enriched in F-HFD. The *y*-axis displays the log-scale LDA scores, highlighting the most significantly different taxa with LDA scores greater than 2 as identified by LEfSe. Red bars indicate taxa with higher relative abundance in the gut microbiota of S-HFD mice, while green bars represent taxa with lower relative abundance in S-HFD compared to F-HFD mice. **(B)** Phylogenetic classification of bacterial populations with LDA scores >2, as well as the subordinate phyla with LDA scores ≤ 2 are displayed. Red denotes higher differential abundance, and green color denotes lower differential abundance microbiota in S-HFD fed mice. **(C)** Heatmap depicting the relative abundance of bacterial phyla across samples from S-HFD and F-HFD groups. Color intensity indicates the standardized abundance values (*Z*-scores), with red representing higher and blue representing lower abundance.

To further identify the key microbial taxa driving differences between the F-HFD and S-HFD groups, we performed LEfSe analysis. The LDA score plot (Figure 3A) revealed several taxa significantly enriched in each group. In the S-HFD group, species such as *Ruminococcus gnavus*, *Staphylococcus sciuri*, *Candida guilliermondii*, and *Clostridium stationis* were among the top discriminant features. In contrast, the F-HFD group was characterized by higher abundances of *Akkermansia muciniphila*, *Coprococcus*, and members of the RF39 group.

A cladogram summarizing the phylogenetic distribution of differentially abundant taxa (Figure 3B) shows distinct lineage-level shifts, with S-HFD enriched in taxa from *Firmicutes*, *Proteobacteria*, and *Tenericutes*, while *Verrucomicrobia* and *RF39* were prominent in the F-HFD group. Heatmap analysis of microbial abundance at the phylum level (Figure 3C) further supports these findings, showing clear differences in microbial signatures between dietary groups. Notably, *Firmicutes* and *Actinobacteria* were more abundant in the S-HFD group, while *Verrucomicrobia* and *TM7* were more represented in F-HFD samples.

To further understand the potential for gut microbiota alteration in S-HFD fed mice, we investigated differences in the composition of gut microbiomes of S-HFD and F-HFD dietary groups. To this end, we used a compositional method to search for distance patterns to identify the correlations of taxa in relation to S-HFD and F-HFD diets. This analysis was performed using LEfSe, which compares relative abundances to identify differentially enriched taxa. Taxa with *p* values less than 0.05 and LDA scores greater than 2 were considered significantly enriched. Compared with F-HFD fed mice, a total of 17 microbiota were significantly changed, among which 14 microbiomes were higher abundance and 3 microbiomes were lower abundance in S-HFD fed mice (Figure 3A). The S-HFD group was predominantly enriched with numerous bacterial taxa, including *Oscillospira guilliemondii*, *Allobaculum*, *Yaniella*, *Ruminococcus gnavus*, *Staphylococcus sciuri*, *Clostridium stationis*, *Corynebacterium*, *Adlercreutzia*, *Aerococcaceae*, *Anaeroplasma*, *Mogibacteriaceae*, *Christensenellaceae*, and *CandidatusArthromitus*, all with LDA scores exceeding 2.0. In contrast, the F-HFD group showed enrichment of only a few taxa, notably *Sutterella*, *Coprococcus*, and *Akkermansia muciniphila*, with negative LDA scores, indicating their relative depletion in S-HFD.

Distinct gut microbial taxa with an LDA score greater than 2, spanning from the phylum to species level, are presented in Figure 3A, along with their corresponding phylogenetic relationships depicted in Figure 3B. In gut microbiota of S-HFD fed mice, expansion of bacterial members in p_Verrucomicrobia, p_ Firmicutes, p_Tenericutes, p_Proteobacteria and p_Actinobacteria were detected (Figure 3B). The heatmap of microbial phylum-level abundance across both diet groups, revealing that Firmicutes, Actinobacteria, Proteobacteria, Deferribacteres, and Tenericutes were more abundant in S-HFD, whereas Verrucomicrobia represented primarily by *Akkermansia muciniphila* was notably elevated in the F-HFD group (Figure 3C).

**Figure 4:**
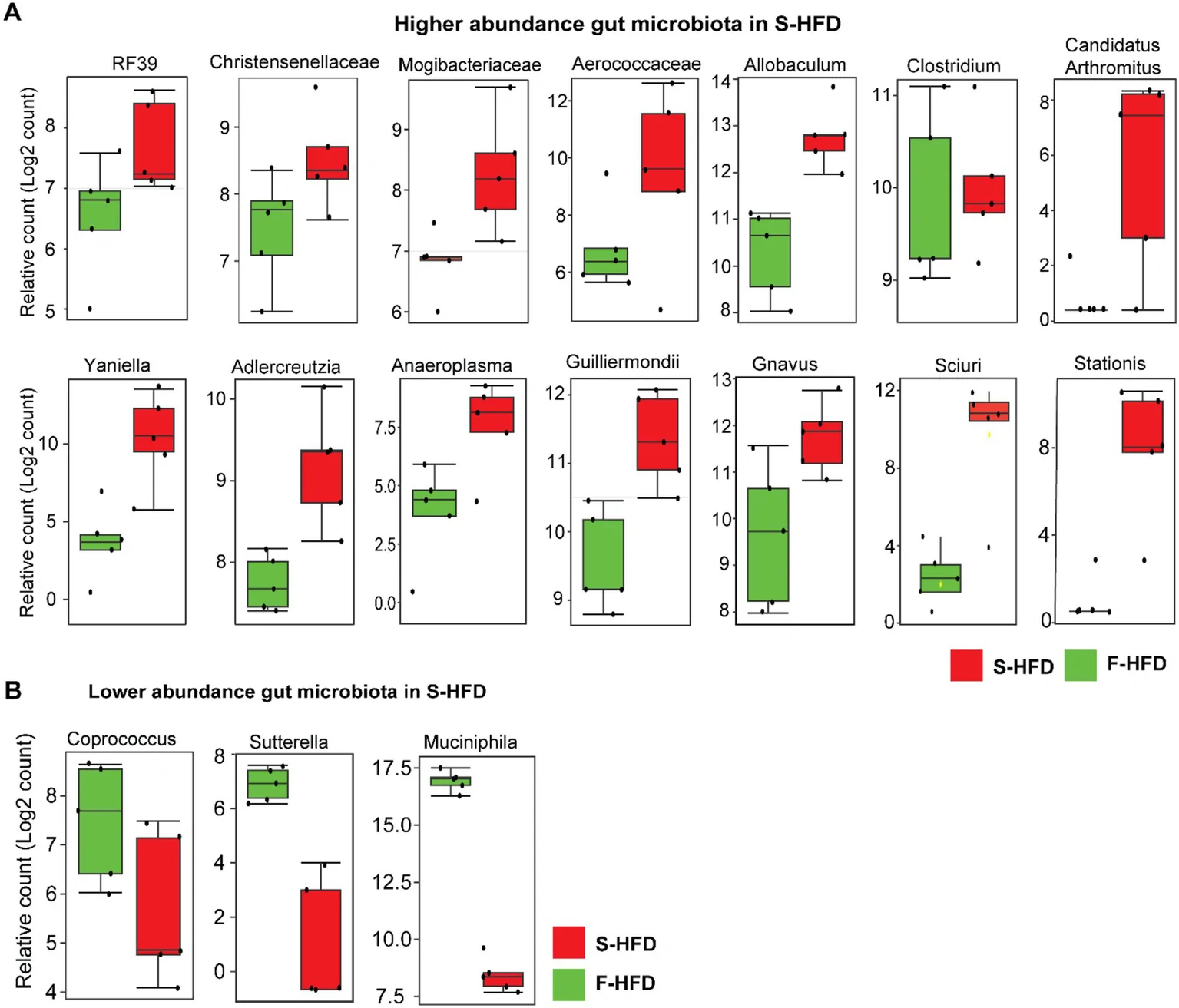
Distribution plots of key microbial taxa identified by LDA score analysis between S-HFD and F-HFD groups. Relative abundance (Log2 count) of significantly discriminant gut microbial taxa identified through Linear Discriminant Analysis (LDA) in Soybean oil-based high-fat diet (S-HFD) and Fish oil-based high-fat diet (F-HFD) groups. **(A)** Taxa with significantly higher abundance in S-HFD. **(B)** Taxa with significantly lower abundance in S-HFD. The horizontal line within each box represents the median, boundaries indicate the interquartile range (IQR), whiskers extend to 1.5 × IQR, and individual data points are shown as dots. Red and green colors correspond to S-HFD and F-HFD groups, respectively.

In the S-HFD group, several gut microbial taxa were enriched, including the class *Mollicutes*, the order *RF39,* family (*Aerococcaceae*, *Mogibacteriaceae*, *Christensenellaceae*) and genus (*Allobaculum*, *Clostridium*, *Candidatus Arthromitus, Yaniella*, *Adlercreutzia* and *Anaeroplasm) and species (guilliermondii*, gnavus, *sciuri* and *stationis*) (Figure 4A). In contrast, the relative abundance of *Coprococcus*, *Sutterella*, and *Akkermansia muciniphila* was reduced in this group (Figure 4B).

### 3.4 Predicted metabolomic profiling of microbial communities

**Figure 5:**
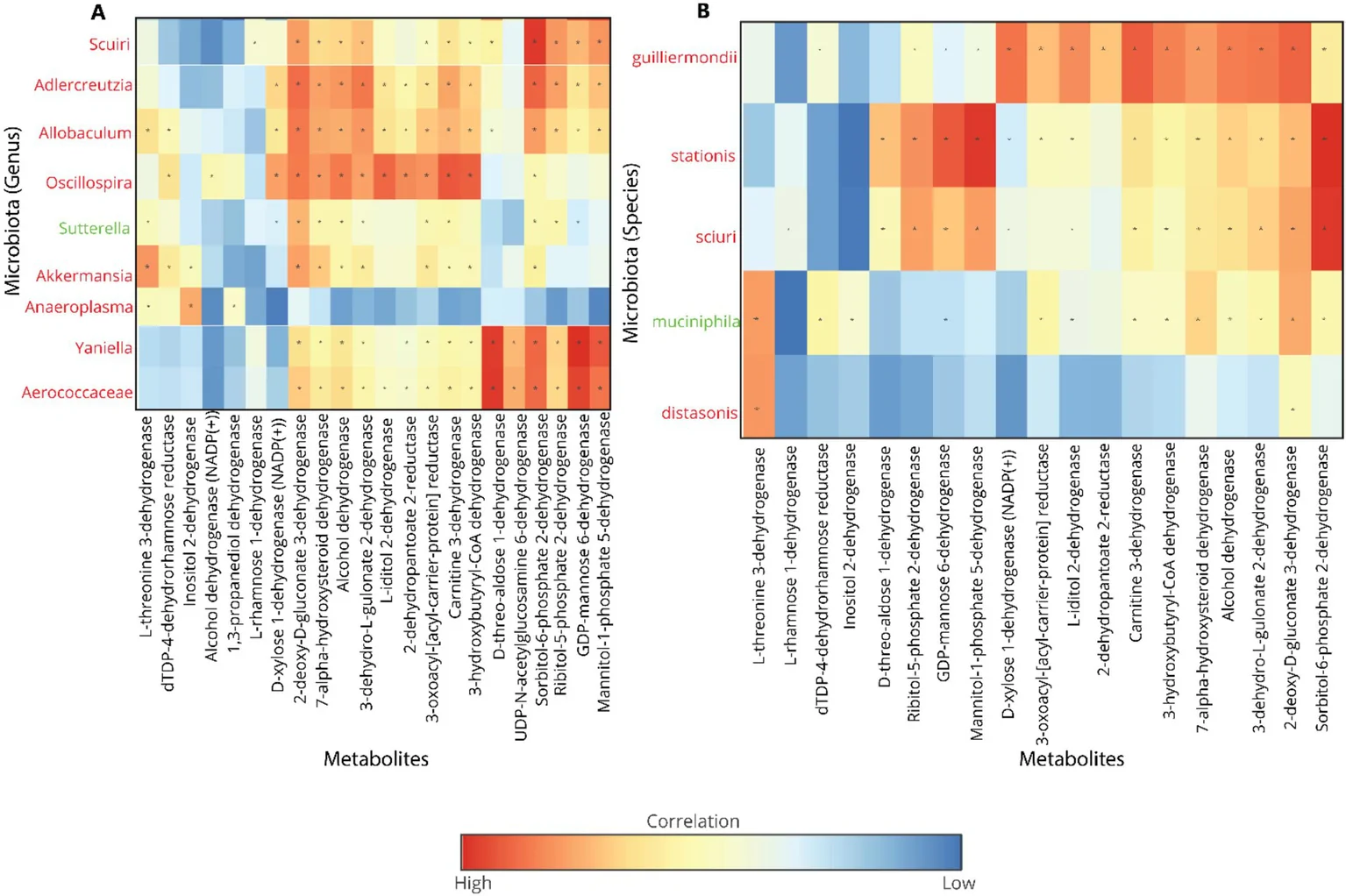
Association between gut microbiota and metabolites from various enzymatic pathways. **(A)** The association between microbial taxa of genus level and metabolites from various enzymatic pathways was assessed by linear regression analysis. **(B)** Heatmap displaying Pearson’s correlation between microbial species and the same set of predicted enzymes. Positive correlations are indicated by red shades, while negative correlations are represented by blue shades, with color intensity reflecting the strength of the correlation. Asterisks (*) denote statistically significant correlations (*p* < 0.05). Microbial taxa enriched in the S-HFD group are labeled in red, while those enriched in the F-HFD group are labeled in green. Correlation analysis was performed using MicrobiomeAnalyst based on predicted functional profiling from PICRUSt2 outputs.

The functional potential of bacterial communities was inferred using PICRUSt2 to predict the abundance of microbial enzymes. The volcano plot and heat map show that the enzymes involved in metabolism in gut are different between F-HFD and S-HFD fed mice, predicting the potential differences of microbial enzyme production in relation to gut microbiomes found in these two dietary groups. Using PICRUSt2, we were able to determine which of the bacterial taxa that we found significantly different contributed to the significant changes in metabolic related enzymes (Figure 5). To explore the relationship between gut microbiota and bacterial enzyme-associated metabolites in S-HFD- and F-HFD-fed mice, Pearson’s correlation analysis was conducted between differentially abundant microbial taxa and predicted metabolic functions using MicrobiomeAnalyst (53). We observed that mice fed the S-HFD had more of certain gut microbes, such as Yaniella, Aerococcaceae, and sciuri stationis. These microbes were strongly associated with various enzyme pathways, including D-threo-aldose 1-dehydrogenase, sorbitol-6-phosphate 2-dehydrogenase, UDP-N-acetylglucosamine 6-dehydrogenase, GDP-mannose 6-dehydrogenase, ribitol-5-phosphate 2-dehydrogenase and mannitol-1-phosphate 5-dehydrogenase (Figures 5A,B). Furthermore, the genera including Oscillospira, Allobaculum and Adlercreutzia, and the species guilliermondii were strongly associated with microbial metabolites from various enzymatic pathways including 2-deoxy-D-gluconate 3-dehydrogenase, 7-alpha-hydroxysteroid dehydrogenase, 3-dehydro-L-gulonate 2-dehydrogenase, Alcohol dehydrogenase, 2-dehydropantoate 2-reductase, 3-oxoacyl-[acyl-carrier-protein] reductase, Carnitine 3-dehydrogenase and 3-hydroxybutyryl-CoA dehydrogenase (Figures 5A,B).

### 3.5 Association of the identified biomarker gut microbiota with host genes

**Figure 6:**
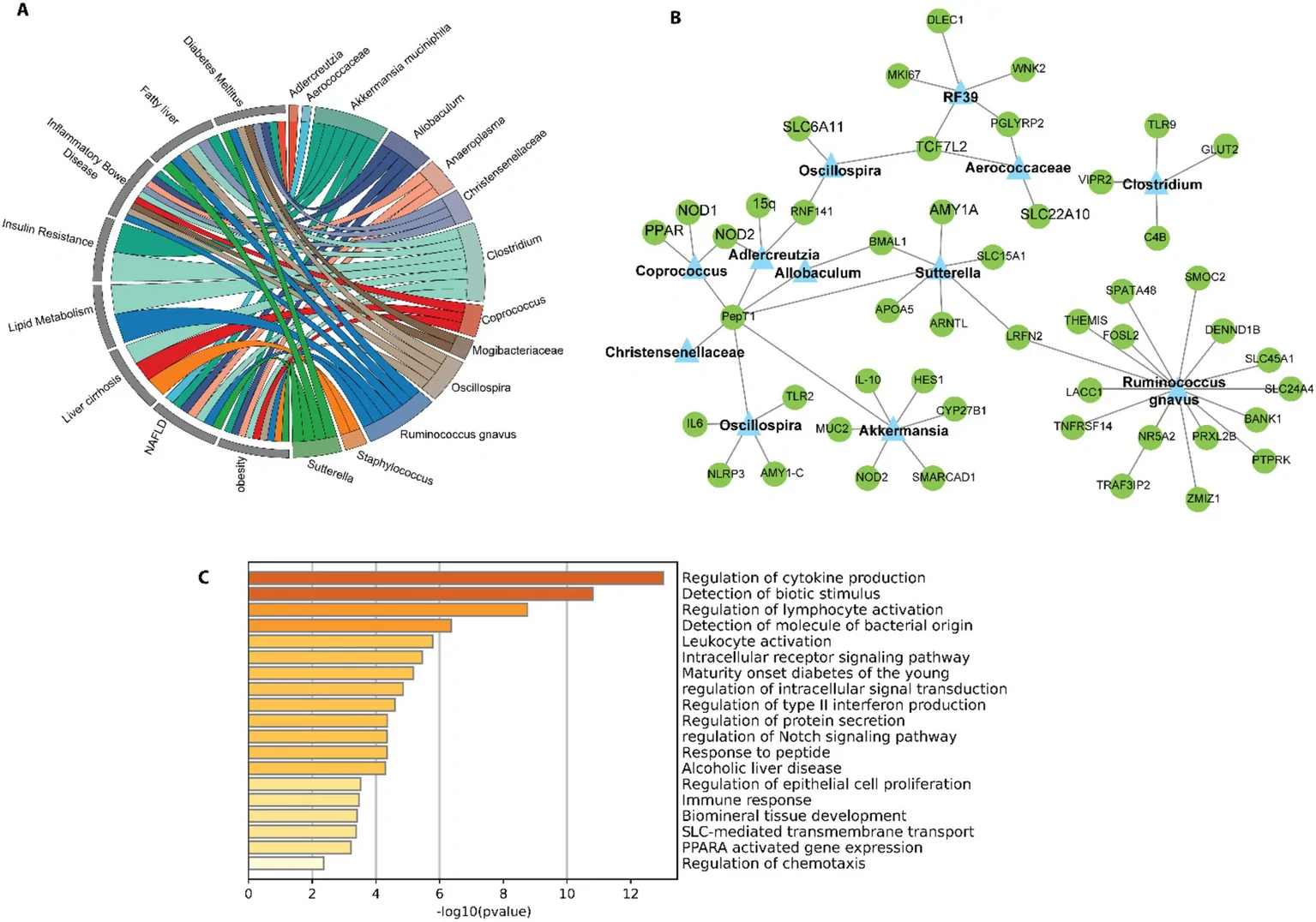
Host-disease associations and functional implications of gut microbiota alterations in S-HFD and F-HFD groups. **(A)** Chord diagram showing reported associations between key microbial taxa and host diseases or metabolic conditions, including obesity, NAFLD, insulin resistance, lipid metabolism, liver cirrhosis, and inflammatory bowel disease, based on microbiota–disease associations curated in the Human Microbiome–Disease Association Database (HMDAD) (see text footnote 2). Connections are based on literature-supported microbial biomarkers and disease relevance. **(B)** Microbiota–host gene interaction network representing reported associations between key microbial genera and host genes involved in immune regulation and metabolic processes, based on the Microbiome-Host Epigenome (MIAOME) database (see text footnote 1). Blue nodes represent microbial taxa, and green nodes represent host genes. Edges indicate predicted functional interactions. **(C)** Bar graph depicting the functional enrichment analysis of host genes associated with differentially abundant microbiota. The most significantly enriched biological processes include cytokine production, lymphocyte activation, immune signaling, metabolic regulation, and inflammation. The *x*-axis shows the -log_10_ (*p*-value) of enrichment significance.

To investigate potential host-pathogen interactions and functional implications of microbial dysbiosis, we performed integrative analysis linking microbial taxa with host diseases, genes, and biological pathways. These predicted associations between biomarker gut microbiota and diseases were identified based on previously reported findings in scientific literature (59). The chord diagram illustrates associations between key gut microbial genera and various metabolic and inflammatory diseases (Figure 6A). Microbes such as *Akkermansia muciniphila*, *Sutterella*, and *Coprococcus* were primarily linked to beneficial functions like lipid metabolism and reduced risk of metabolic disorders, while taxa enriched in the S-HFD group, including *Ruminococcus gnavus*, *Staphylococcus*, and *Mogibacteriaceae*, were associated with obesity, NAFLD, insulin resistance, and inflammatory bowel disease.

Microbe-gene network further demonstrated the connectivity between specific microbial genera and host genes involved in immune regulation and metabolic processes. For instance, *Oscillospira* and *Akkermansia* were associated with anti-inflammatory genes such as *IL10*, *NOD2*, and *TLR2*, whereas *R. gnavus* and *Clostridium* were linked to pro-inflammatory and metabolic dysregulation-related genes, including *TNFRSF14*, *SMOC2*, and *SLC24A1* (Figure 6B). Gene Ontology (GO) enrichment analysis of host genes associated with microbial taxa revealed significant enrichment in immune-related processes, including regulation of cytokine production, lymphocyte activation, leukocyte response, and intracellular signaling pathways (Figure 6C). Notably, pathways related to metabolic diseases such as maturity onset diabetes of the young and alcoholic liver disease were also significantly enriched. These findings indicate that microbiota altered under S-HFD feeding are linked, based on predictive and literature-supported analyses, to host immune and metabolic pathways relevant to inflammation and metabolic disease.

### 3.6 Mice fed S-HFD displayed liver steatosis and inflammation

**Figure 7:**
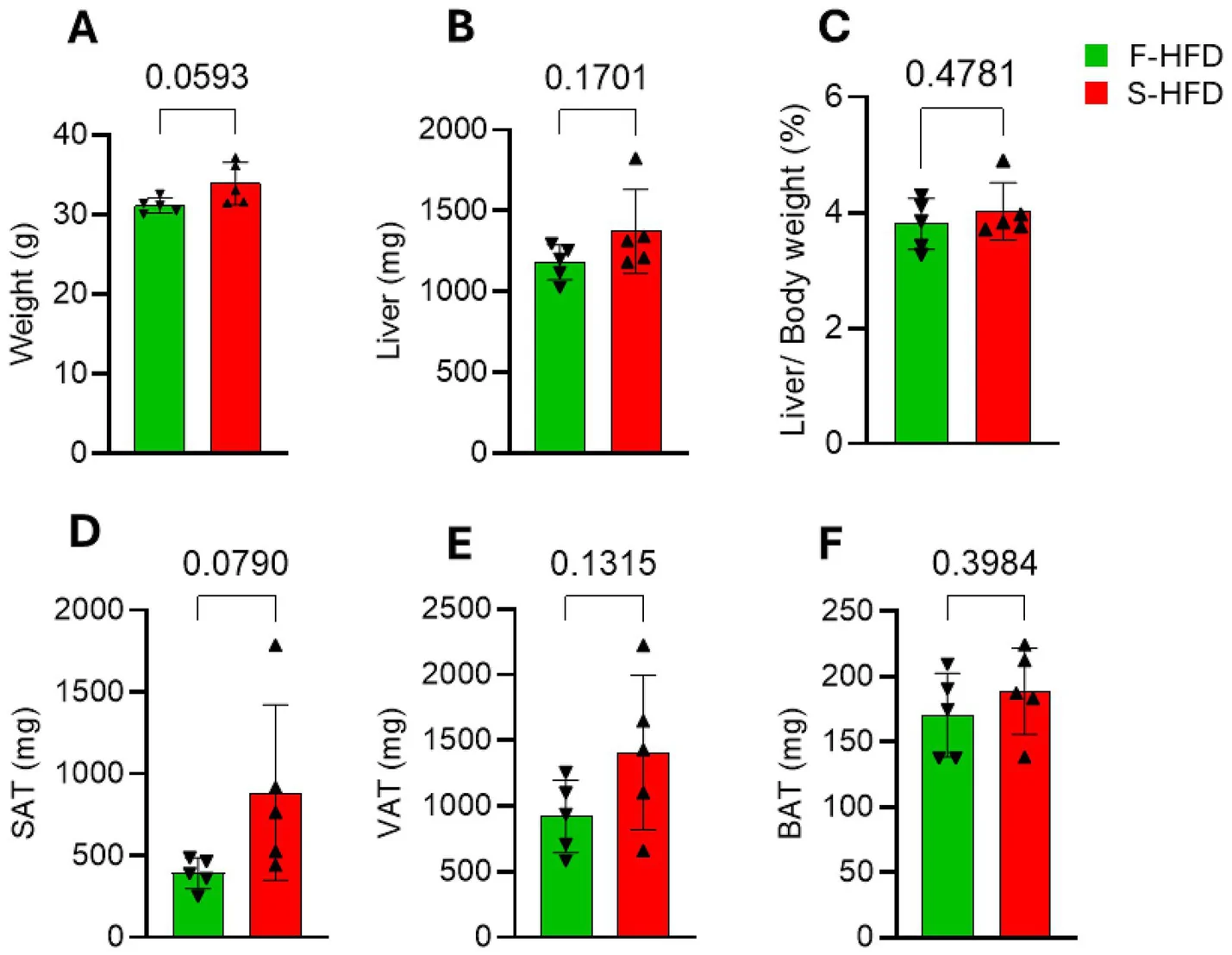
Comparison of body and tissue weights in mice fed S-HFD and F-HFD for 24 weeks. **(A)** After 24 weeks of dietary intervention with S-HFD or F-HFD, body weights of the mice (*n* = 5–6 per group) were recorded. Mice were then sacrificed for tissue collection and further analysis. **(B,C)** Liver weight and liver-to-body weight ratio (expressed as a percentage). **(D–F)** The weights of subcutaneous adipose tissue (SAT), visceral adipose tissue (VAT), and brown adipose tissue (BAT) were measured in mice. Green bars represent the Fish oil-based diet (F-HFD), while red bars represent the Sunflower oil-based diet (S-HFD). Data are shown as means ± SEM. *p-value ≤ 0.05.

In this study, over a 24-week period, we assessed and compared the final body and tissue weights of mice receiving either a sunflower-fat diet (S-HFD) or a fish-fat diet (F-HFD). The analysis revealed no notable differences between the two groups in terms of total body weight, or in the weights of brown adipose tissue (BAT), inguinal subcutaneous fat (SAT), epididymal visceral fat (VAT), or liver tissue (Figure 7).

**Figure 8:**
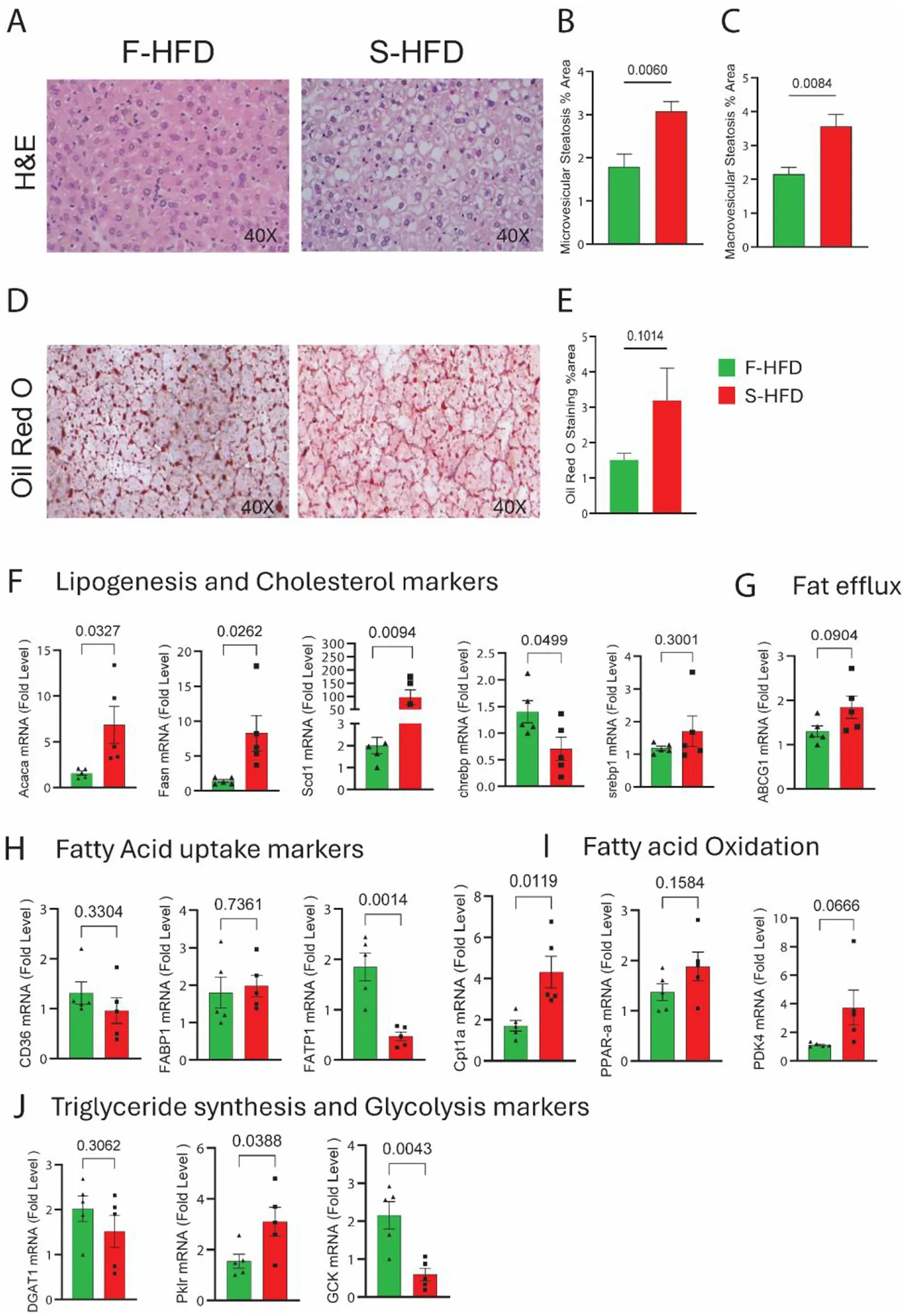
The sucrose-free S-HFD induces hepatic steatosis and activates the full lipogenic program. **(A)** Liver sections from mice (*n* = 5–6 per group) fed F-HFD or S-HFD for 24 weeks were stained with hematoxylin and eosin (H&E) and examined at 20 × original magnification. **(B,C)** The percentages of microvesicular and macrovesicular steatosis were quantified to assess the extent of hepatic fat accumulation. **(D,E)** Lipid deposition was assessed by Oil Red O staining of liver tissues, with the percentage of stained area used as a measure of lipid content. **(F)** mRNA expression of genes related to *de novo* lipogenesis (*Acaca, Fasn, Scd1*) and cholesterol synthesis (*Srebp1, Chrebp*). **(G)** Expression of the fat efflux–related gene *Abcg1* was measured. **(H)** Genes associated with fatty acid uptake (*CD36, Fabp1, Fatp1*) were also evaluated. **(I)** Expression of genes involved in *β*-oxidation (*Cpt1a, Ppara, PDK4*) was assessed. **(J)** mRNA expression of genes related to triglyceride synthesis and glycolysis markers (*DGAT1, Pklr, GCK*) were measured. The green bar represents fish diet, red bar represents sunflower diet. Data are shown as means ± SEM. *p-value ≤ 0.05.

To investigate whether the alterations in gut microbiota induced by the S-HFD diet affected liver function, we performed histological analysis of liver tissues. As shown in Figure 8, livers from S-HFD-fed mice exhibited both microvesicular and macrovesicular steatosis, indicative of lipid accumulation. In contrast, no visible signs of hepatic fat accumulation were observed in the F-HFD group (Figures 8A–D). These observations were further supported by Oil Red O staining, which confirmed substantial lipid deposition in the livers of S-HFD-fed mice, while minimal staining was observed in the F-HFD group (Figures 8D–E). These findings suggest that S-HFD feeding is associated with hepatic steatosis and occurs alongside marked alterations in gut microbiota composition.

In line with steatosis development, liver gene expression analysis from mice fed the S-HFD revealed a significant increase in the expression of genes associated with lipid accumulation including: (i) *de novo* lipogenesis (*Acaca, Fasn,* and *Scd1*) (Figure 8F), and (ii) Glycolysis (*Pklr*) as compared with expression of these target mRNAs in liver samples from F-HFD fed mice (Figure 8J), whereas no significant difference was found in the *Srebp1*, *Chrebp* (Figure 8F) and for fat efflux gene there was no significant difference seen in the expression of *Abcg* (Figure 8G). Moreover, no significant differences were observed in the hepatic expression of the fatty acid transporter *Cd36* or *Fabp1* between the two dietary groups. However, the expression of long-chain *Fatp1* was notably upregulated in the livers of F-HFD-fed mice (Figure 8H). In terms of *β*-oxidation-related genes, *Cpt1a* expression was significantly increased in the S-HFD group, while *Ppara* and *Pdk4* transcripts showed a modest upregulation compared to the F-HFD group (Figure 8I). The mRNA expression for *DGAT1*, a gene involved in triglyceride synthesis, was similar in the two groups. mRNA for the glycolysis marker *GCK* was significantly downregulated in S-HFD compared to F-HFD (Figure 8J).

**Figure 9:**
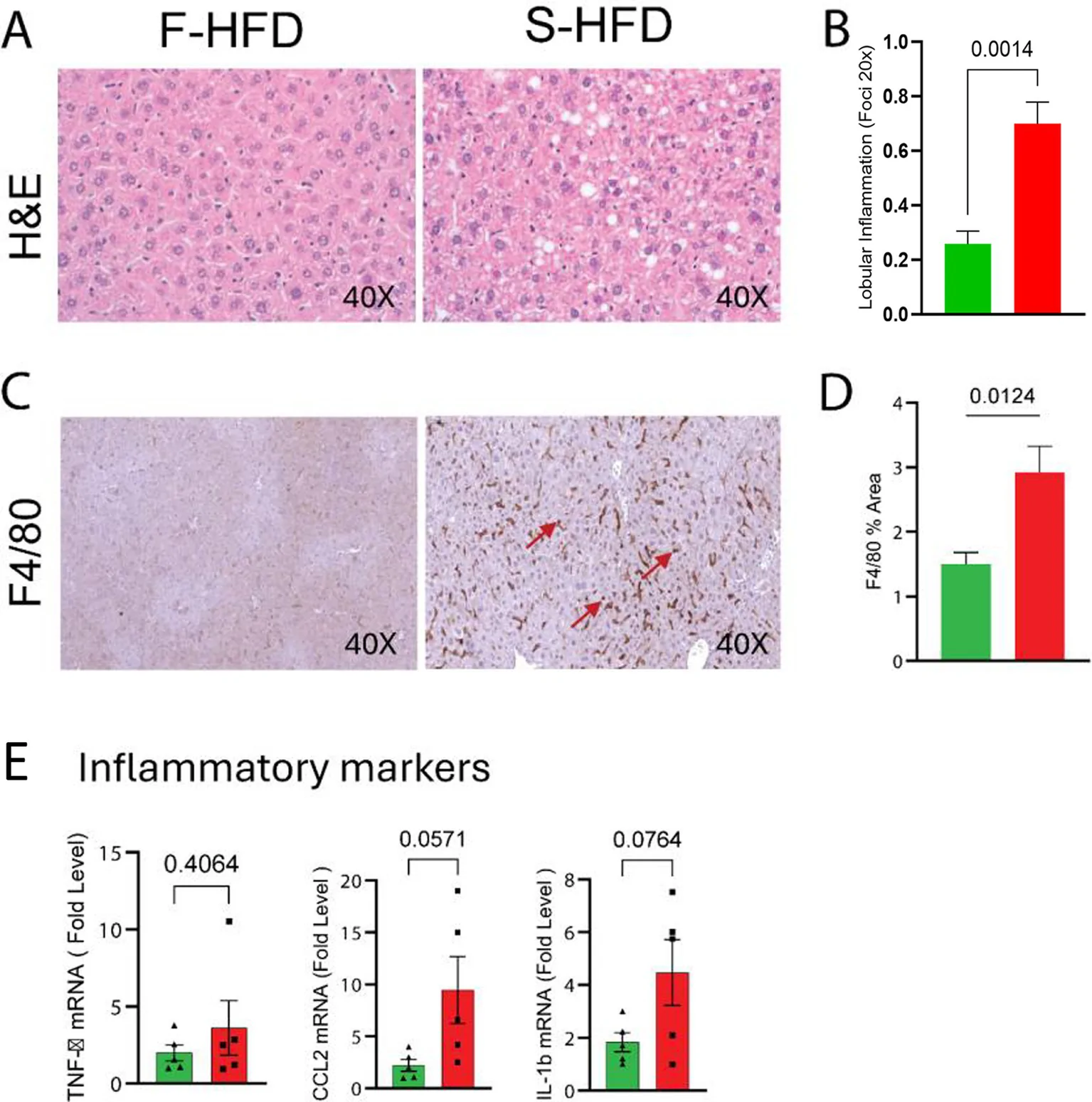
S-HFD induces hepatic inflammation in mice. Representative hematoxylin and eosin (H&E) stained liver sections from mice fed F-HFD **(A)** or S-HFD **(B)** for 24 weeks, shown at 20 × and 40 × magnification. Red arrows in S-HFD livers indicate areas of lobular inflammation. **(C)** Hepatic immune cell infiltration was assessed by immunohistochemical staining for the macrophage marker F4/80. **(D)** F4/80% area. **(E)** mRNA expression levels of inflammation-related genes (*Tnf-α*, *Ccl2*, and *Il-1β*) were quantified by real-time RT-qPCR. Green bars represent the fish oil-based diet (F-HFD), and red bars represent the sunflower oil-based diet (S-HFD). Data are expressed as mean ± SEM. Statistical significance was determined using an unpaired Student’s *t*-test, with *p*-value < 0.05 considered significant.

We next evaluated hepatic inflammation in mice fed F-HFD or S-HFD. Histopathological analysis revealed prominent lobular inflammation in the livers of S-HFD-fed mice (Figures 9A,B). Additionally, immunostaining showed an increased accumulation of F4/80-positive macrophages in the liver tissues of the S-HFD group, indicating enhanced inflammatory cell infiltration (Figures 9C,D). In line with these findings, the expression of key inflammatory markers, including *Tnf-α*, *Ccl2* (also known as *Mcp-1*), and *Il-1β*, was significantly upregulated in the livers of S-HFD-fed mice compared to those fed the F-HFD (Figure 9E). These results collectively indicate that S-HFD feeding is associated with hepatic inflammation and increased macrophage accumulation.

#### 3.6.1 Glucose tolerance, insulin resistance, and circulating metabolic hormones

**Figure 10:**
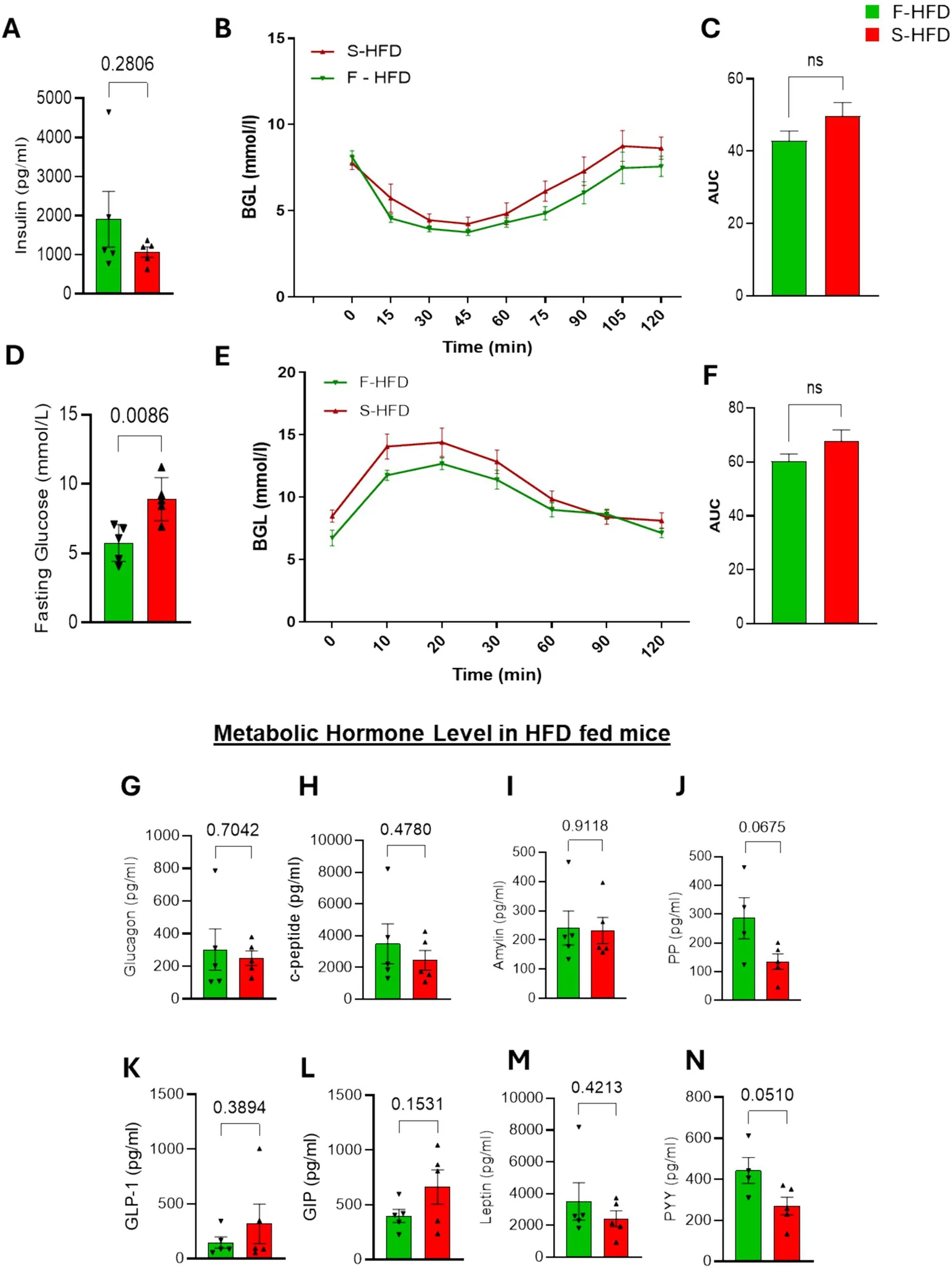
The glucose tolerance, insulin resistance, and metabolic hormone levels of mice fed S-HFD and F-HFD. **(A)** Fasting plasma insulin levels were measured. **(B)** Insulin tolerance test (ITT) was performed after 21 weeks; blood glucose levels were monitored over time. **(C)** AUC for comparing insulin sensitivity. **(D)** Fasting blood glucose was measured at 22 weeks of dietary intervention. **(E)** OGTT was performed after 22 weeks; blood glucose levels were plotted over time. **(F)** AUC for OGT, comparing glucose tolerance between groups **(G–N)** Plasma concentrations of key metabolic markers were determined in fasting mice using Milliplex multiplex assay kits. Green bars represent the F-HFD, and red bars represent the S-HFD. All values are expressed as mean ± SEM. Statistical significance was assessed using Student’s *t-*test; *p* < 0.05, *p* < 0.01.

Compared to the F-HFD group, fasting insulin levels were trending to be lower in the S-HFD group (Figure 10A). Insulin tolerance test (ITT) revealed that blood glucose levels decreased for both S-HFD and F-HFD, and the area under the curve did not show any significant difference between groups (Figures 10B,C). We also observed significant differences in the fasting blood glucose levels in plasma samples from S-HFD-fed mice compared to those from F-HFD fed mice (Figure 10D). Furthermore, OGTT analysis revealed no significant change in glucose tolerance in S-HFD-fed mice compared to F-HFD fed mice (Figures 10E,F).

We compared the plasma levels of metabolic hormones in both groups. As shown in (Figures 10G–N), the levels of glucagon, C-peptide, amylin, leptin, GIP, GLP, PP and peptide YY (PYY) were not significantly different in S-HFD-fed mice and F-HFD-fed mice.

## 4 Discussion

This study identifies clinic-metabolic and gut microbiome differences between mice fed with high-fat diets from two different sources: fish and sunflower oils (F- and S-HFD, respectively). Although no significant differences were found between the two groups regarding body weight gain, the liver histopathology revealed increased macrovascular steatosis and lobular inflammation in mice fed on S-HFD compared with mice fed with F-HFD. Furthermore, the S-HFD fed group had increased hepatic macrophage infiltration. Fish oil is prized for its high *ω*-3 fatty acids content such as eicosapentaenoic acid (EPA) and docosahexaenoic acid (DHA), whereas sunflower oil is largely valued for its *ω*-6 fatty acids content (61). A diet excessively high in ω-6 and low in ω-3 fatty acids may cause an imbalance and predispose to inflammatory responses, perpetuating chronic low-grade inflammation (62). This is expected as ω-6 fatty acids, especially the linoleic acid (18:2n-6), are converted into arachidonic acid, leading to synthesis of proinflammatory derivatives including prostaglandins, leukotrienes, and thromboxanes (63), all of which may lead to chronic liver inflammation and hepatic fat accumulation (64). Our findings are supported, at least in part, by others reporting that ω-6 fatty acid intake may lead to liver inflammation, oxidative stress, and fibrosis (65).

Regarding metabolic differences, the S-HFD group displayed higher fasting blood glucose levels, compared to F-HFD group. Given that chronic feeding of a HFD rich in ω-6 fatty acids may impair insulin sensitivity (66), relatively higher blood glucose levels in S-HFD mice compared to F-HFD mice are not unexpected. Notably, ω-6 fatty acids may enhance insulin secretion and/or reduce its catabolism, causing impairment in insulin action and development of insulin resistance (67, 68), suggesting a reciprocal link between ω-6 fatty acids pathway and insulin secretion (66).

Unlike F-HFD fed mice, S-HFD fed mice developed steatosis which was consistent with the increased hepatic gene expression of lipogenic markers (*Acaca*, *Fasn*, and *Scd1*) in these mice. Regarding fatty acid uptake markers, both groups had comparable expression levels of *Cd36* and *Fabp1*, while *Fatp1* expression was downregulated in S-HFD mice. Taken together, it implies that hepatic lipogenesis might have been a major player in steatosis observed in S-HFD fed mice. These findings can be explained based on the evidence that intake of ω-6 fatty acids rich diets led to upregulated expression of *SREBP-1c* transcription factor in the liver which promoted lipid synthesis (69). Our finding of diminished hepatic expression of *Chrebp* in S-HFD fed mice points to a possibility that the intake of ω-6 rich S-HFD could lead to steatosis in mice via a mechanism independent of the conversion of carbohydrates into fat in the liver (70, 71). Although the hepatic expression of *Cpt1a* was significantly increased in S-HFD mice compared to F-HFD mice, the expression of other fatty acid metabolism markers (*Ppara* and *Pdk4*) did not differ significantly between two groups, implying that the lipid transport and metabolism could not have been differentially affected by these two dietary fats. Overall, the fatty acid metabolism markers that we tested in this study trended for a relatively higher expression in S-HFD mice than F-HFD mice. In line with our findings, sunflower HFD administration in mice was found to increase the expression of fatty acid *β*-oxidation-related genes, including *Cpt1a* (72).

Among triglyceride synthesis and glycolysis markers, *Pklr* expression was elevated while *Gck* expression was suppressed in S-HFD mice than F-HFD mice, whereas the *Dgat1* expression differed non-significantly between two groups. The increased *Pklr* with reduced *Gck* expression in the livers of S-HFD mice suggests a metabolic shift, pointing to insulin resistance in these mice. The S-HFD mice also showed increased hepatic inflammation as indicated by increased *Ccl2* expression in the liver which is a known marker for monocytic infiltration. Commensurate with these changes, S-HFD mice had significantly increased fasting plasma glucose levels, showing impaired glycemic control. Our findings are supported, at least in part, by a previous study showing development of insulin resistance in mice, indicated by reduced plasma membrane expression of *Glut4*, following daily subcutaneous injections of sunflower oil for 7 days (18). Similarly, the reduced hepatic *Gck* expression that we observed in S-HFD mice is consistent with their glucose intolerance and insulin resistance attributes since a negative correlation of hepatic *Gck* expression with HbA1c and fasting glucose was observed in patients with type 2 diabetes (73), whereas the *Gck* activation promoted glucose tolerance and insulin sensitivity but exacerbated hepatic lipid accumulation in mice (74). In line with increased hepatic *Ccl2* expression in S-HFD mice, as expected, we found infiltration of F4/80-positive cells in the livers of these mice, unlike F-HFD mice, suggesting that feeding of S-HFD might promote a proinflammatory milieu in the liver. Likewise, in previous studies sunflower oil administration led to increased proinflammatory marker (TNF-*α* and IL-6) expression in the peripheral blood mononuclear cells of individuals with morbid obesity (75). Thus, the differential effects of dietary fats on liver health underscore the importance of balancing *ω*-3 and ω-6 fatty acid intake to mitigate inflammatory and metabolic risks.

The gut microbiome plays an important role in metabolic regulation in the host and an imbalance of microbial communities or induction of dysbiosis can impact a wide range of metabolic responses in the host. In this regard, differential microbiome changes were observed between mice fed with S-HFD or F-HFD. Notably, *Firmicutes* were highly abundant (40%) in S-HFD fed mice compared with F-HFD fed mice (3%), whereas *Verrucomicrobia* dominated in F-HFD fed mice compared with S-HFD fed mice (<1%). *Firmicutes of the* species including *Ruminococcaceae*, *Lachnospiraceae*, and *Lactobacillaceae* contribute to dietary fiber fermentation into short-chain fatty acids (SCFAs) which maintain gut health and influence systemic metabolism (76). Nonetheless, the gut dysbiosis marked by an overabundance of *Firmicutes* may have metabolic consequences, such as dysregulated lipid metabolism, obesity, increased inflammation, and insulin resistance (77, 78). These effects are largely mediated by induced dysregulation at multiple levels involving energy extraction from the diet, altered SCFA production, disruption of the gut barrier, and impaired immune functions. On the other hand, *Verrucomicrobia*, which we found as dominant gut microbiota in F-HFD fed mice, are emerging as important players in metabolic homeostasis. This phylum, especially the species *Akkermansia muciniphila*, are crucial for metabolic health, by reducing inflammation, improving gut barrier integrity, and maintaining insulin sensitivity (79). These species also maintain gut health and systemic metabolism by regulating weight, lipid metabolism and production of SCFAs (80, 81).

The phylum *Bacteroidetes* have the potential to degrade complex polysaccharides such as dietary fibers, resistant starches, cellulose, pectin, xylan, and even non-digestible carbohydrates, produce beneficial SCFAs, as well as regulate immune responses. Thus, they act as key players in maintaining energy balance, gut health, and metabolism of the host. Of note, the *Firmicutes*/*Bacteroidetes* (F/B) ratios in S-HFD and F-HFD mice were 1.32 and 2.45, respectively. The decreased F/B ratio in S-HFD fed mice indicates that in addition to promoting growth of *Firmicutes*, sunflower oil also promoted the growth of beneficial *Bacteroidetes*. Importantly, since the *Firmicutes* versus *Bacteroidetes* interactions in the gut are complex, the moderately increased F/B ratio following F-HFD does not necessarily equate to negative metabolic effects. This may be due to compensation by the beneficial effects of *ω*-3 fatty acids reducing inflammation and improving metabolic function, these benefits are widely recognized (82–85). Overall, sunflower oil-based HFD feeding was associated with a dysbiotic gut microbiota profile marked by the higher abundance of opportunistic commensal gut microbiota (*Clostridium, Allobaculum, Yaniella, C. guilliermondii, R. gnavus, A. stationis, Adlercreutzia, Aerococcaceae, Anaeroplasma, Mogibacteriaceae,* and *Candidatus_Arthromitus*) and the lower abundance of beneficial microbial taxa (*Muciniphila, Sutterella,* and *Coprococcus*). In the metabolic context, beneficial potential and protective roles of *A. muciniphila, Sutterella,* and *Coprococcus* are well documented (86–88). It is noteworthy that the highly abundant microbial taxa detected in S-HFD fed mice predictively associated with the host genes linked to metabolic disorders and pathways of inflammation and immune dysregulation. In concordance, S-HFD fed mice showed a peculiar immune metabolic phenotype marked by increased fasting blood glucose, and increased F4/80 + cells in the liver and inflammation, steatosis. In terms of gene expression, we observed higher expression of genes associated with fatty acid uptake, *de novo* lipogenesis, fatty acid oxidation, glycolysis, and monocyte chemotaxis. Nonetheless, our study remains limited by certain caveats as we could not show the causal relationship between gut dysbiosis bacteria and steatohepatitis in S-HFD fed mice. Moreover, the number of mice in each dietary intervention was relatively small. We will address these aspects in future investigations. In conclusion, we herein show that S-HFD feeding was associated with gut dysbiosis in mice, accompanied by pathophysiologic features consistent with moderate steatohepatitis and loss of glycemic control.

A key limitation of this study is that it demonstrates associations rather than direct causality between gut microbiota alterations and liver pathology. Although S-HFD feeding was accompanied by gut dysbiosis, hepatic steatosis, macrophage infiltration, inflammatory gene expression, and altered metabolic pathways, the current study design does not establish whether microbiota changes directly drive steatohepatitis-like features. Future mechanistic studies using fecal microbiota transplantation, antibiotic-mediated microbiota depletion, germ-free mouse models, or targeted supplementation of candidate taxa will be required to determine whether the identified microbial alterations play a causal role in hepatic inflammation and steatosis.

In conclusion, our findings show that sucrose-free S-HFD feeding is associated with a distinct dysbiotic gut microbiota profile, hepatic steatosis, macrophage infiltration, inflammatory gene expression, and impaired fasting glycemia. While these results support a link between dietary fat source, microbiome remodeling, and liver metabolic dysfunction, they do not establish direct causality. Future mechanistic studies are required to determine whether the observed microbiota alterations directly contribute to the development of steatohepatitis-like features.

## Data Availability

The datasets presented in this study can be found in in the NCBI under BioProject accession PRJNA1215797: https://www.ncbi.nlm.nih.gov/bioproject/PRJNA1215797.

## Footnotes

1
https://miaome.idrblab.net/ttd/

2
https://www.cuilab.cn/hmdad
