# Supplementary material for: Gut dysbiosis induced by sunflower oil-based sucrose-free high-fat diet leads to steatohepatitis
Source: Front Nutr. 2026 Sep 11;13:1831803. doi: 10.3389/fnut.2026.1831803 (PMC13614012; doi:10.3389/fnut.2026.1831803)
Supplement: Supplementary file 1 [file Supplementary_file_1.docx]

**Supplementary Table S1:** Primer List

| **Gene Name** | **Assay ID** | **Source** |
| --- | --- | --- |
| Acaca | Mm01304258_m1 | Thermo Fisher Scientific |
| Fasn | Mm00662319_m1 | Thermo Fisher Scientific |
| Scd1 | Mm00772290_m1 | Thermo Fisher Scientific |
| Chrebp | Mm02342723_m1 | Thermo Fisher Scientific |
| Pdk4 | Mm01166879_m1 | Thermo Fisher Scientific |
| DGAT1 | Mm00515643_m1 | Thermo Fisher Scientific |
| Srebp-1 | Mm00550338_m1 | Thermo Fisher Scientific |
| CD36 | Mm00432403_m1 | Thermo Fisher Scientific |
| Fabp1 | Mm00444340_m1 | Thermo Fisher Scientific |
| Pklr | Mm00443090_m1 | Thermo Fisher Scientific |
| Fatp1 | Mm00449511_m1 | Thermo Fisher Scientific |
| Abcg1 | Mm00437390_m1 | Thermo Fisher Scientific |
| GCK | Mm00439129_m1 | Thermo Fisher Scientific |
| Cpt1a | Mm01231183_m1 | Thermo Fisher Scientific |
| Ppara | Mm00440939_m1 | Thermo Fisher Scientific |
| TNF alpha | Mm00443258_m1 | Thermo Fisher Scientific |
| CCL2 | Mm00441242_m1 | Thermo Fisher Scientific |
| IL-1b | Mm00434228_m1 | Thermo Fisher Scientific |
| Gapdh | Mm99999915_g1 | Thermo Fisher Scientific |

**Supplementary Table S2:** Adapter List

| **Forward primer** | TCGTCGGCAGCGTCAGATGTGTATAAGAGACAGCCTACGGGNGGCWGCAG |
| --- | --- |
| **Reverse primer** | GTCTCGTGGGCTCGGAGATGTGTATAAGAGACAGGACTACHVGGGTATCTAATCC |
